# Association between sun-protective behaviors and hypertension: a cross-sectional study from National Health and Nutrition Examination Survey 2009 to 2014

**DOI:** 10.1186/s12889-023-16439-4

**Published:** 2023-09-26

**Authors:** Shuping Yang, Feng Dai, Zhaokai Wang, Ruoshui Li, Xianzhi Xu, Cheng Li, Xiancun Hou, Yang Liu, Chaofan Wang, Dongye Li, Lei Li, Tongda Xu

**Affiliations:** 1 Department of General Practice, The Affiliated Hospital of Xuzhou Medical University, Xuzhou, Jiangsu 221000 People’s Republic of China; 2 Department of Cardiology, The Affiliated Hospital of Xuzhou Medical University, Xuzhou, Jiangsu 221000 People’s Republic of China; 3 Department of Stomatology, The Affiliated Hospital of Xuzhou Medical University, Xuzhou, Jiangsu 221000 People’s Republic of China; 4 Department of Nuclear Medicine, The Affiliated Hospital of Xuzhou Medical University, Xuzhou, Jiangsu 221000 People’s Republic of China; 5 Institute of Cardiovascular Disease Research, Xuzhou Medical University, Xuzhou, Jiangsu 221000 People’s Republic of China

**Keywords:** Hypertension, NHANES, Sun-protective behaviors

## Abstract

**Background:**

In previous studies, sun-protective behaviors increased cardiovascular incidence. Our present article is to further analyze the potential relationship between sun-protective behaviors (staying in the shade, wearing long-sleeved clothing, and applying sunscreen) and hypertension.

**Method:**

The present cross-sectional study evaluated 8,613 participants (aged 20–60 years) from the National Health and Nutrition Examination Survey (NHANES) obtained between 2009 and 2014. We performed multiple logistic regression analysis to examine the relationship between sun-protective behaviors and hypertension. Subgroup analysis was then performed. Multiple linear regression analysis was utilized to examine the relationship of sun-protective behaviors and each sun-protective behavior with systolic and diastolic blood pressure, stratified by sex and race.

**Results:**

A total of 8,613 participants (weighted *n* = 127,909,475) were applied in our study, including 1,694 hypertensive subjects. Our study demonstrated that sun-protective behaviors of the 2–3 category were associated with increased risk of hypertension, but not with higher systolic and diastolic blood pressure. In subgroup analysis, men, Mexican American, and 25 < BMI ≤ 30 who reported sun-protective behaviors (2–3) were prone to hypertension. Multiple linear regression models showed that non-Hispanic white men with sun-protective behaviors (2–3) were positively associated with systolic and diastolic blood pressure. The association between other-Hispanic men with frequent wearing long-sleeved clothing and diastolic blood pressure was positively correlated.

**Conclusion:**

Sun-protective behaviors of the 2–3 category could increase the incidence of hypertension, but not increase systolic and diastolic blood pressure. We only found that non-Hispanic white men who reported sun-protective behaviors (2–3) were positively associated with systolic and diastolic blood pressure. These findings suggested that excessive sun-protective behaviors should be avoided.

**Supplementary Information:**

The online version contains supplementary material available at 10.1186/s12889-023-16439-4.

## Background

Hypertension, defined as measured systolic pressure of ≥ 140 mmHg and diastolic pressure of ≥ 90 mmHg, is a major health problem that traditionally gives rise to atherosclerotic cardiovascular diseases (CVDs) [1]. It commonly coexists with other cardiovascular risk factors and may damage the structures and functions of essential organs, such as the heart, brain, and kidneys, and eventually contribute to their failure [2]. The prevalence and incidence of hypertension vary by country, region, race, and age, and are higher in industrialized countries than in developing countries [3]. Statistical data indicate that in recent three decades, the world has witnessed an increase in hypertension in adults aged 30–79 years from 650 million to 1.28 billion individuals [4]. In 2019, high blood pressure was a major or contributing cause of more than 500,000 deaths in the United States [5]. Hypertension has now become the primary risk factor for cardiac death in the United States [6].

Many studies have found that sunlight provides significant benefits [7]. Sunlight exposure can stimulate vitamin D3 synthesis in the skin [8], which can promote growth and development in children, as well as decrease the risk of osteoporosis, osteopenia, and fractures in adults [9]. It is well documented that people with the greatest sun exposure have a lower cardiovascular mortality risk in comparison to those with inadequate sun exposure [10] and that increased sun exposure in the summer reduces the incidence of myocardial infarction [11]. And as the world population continues to grow, the proportion of indoor workplaces and living increases every year and many people emphasize sun protection [12]. However, many articles only studied the relationship between sunlight and hypertension. Rostand et al. found that people residing in low latitudes had a lower incidence of hypertension because they were exposed to higher rates of solar radiation [13] and Ke et al.confirmed that more sun exposure reduced the risk of hypertension in population from a Macau [14]. Fewer studies have used the direction of sun protection to explore the relationship with hypertension.

Therefore, the present study investigated a potential connection between sun-protective behaviors and hypertension by analyzing adult (aged 20–60 years) patient data from the National Health and Nutrition Examination Survey (NHANES) from 2009–2014.

## Methods

### Data source and study population

**Fig. 1:**
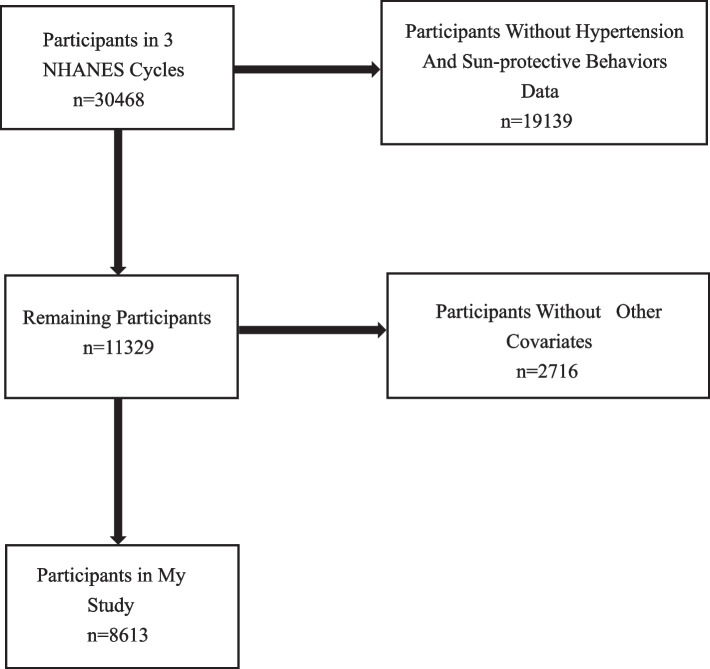
Flowchart of the systematic selection process

Statistical data from the NHANES were analyzed to investigate the relationship between sun-protective behaviors and hypertension. The NHANES is major research study that was carried out by the National Center for Health Statistics, the Centers for Disease Control and Prevention, to assess the state of health and nutrition in the U.S. population. The present study received approval from The National Center for Health Statistics and written informed consent was obtained from all participants. More information is available in the NHANES database, http://www.cdc.gov/Nhanes. A total of 8,613 individuals were included in the study after excluding patients with missing information on sun-protective behaviors, hypertension, and other covariates. The flow chart of the systematic selection process is illustrated in Fig. 1.

### Outcome variable

Blood pressure measurements were obtained by professionally trained personnel. Participants with hypertension were informed by their physicians that they conformed to the hypertension criteria. The criteria are systolic blood pressure ≥ 140 mmHg and diastolic blood pressure ≥ 90 mmHg in three blood pressure measurements taken at the same time on a non-same day.

### Sun-protective behaviors

Sun-protective behavior was assessed using three variables that included staying in the shade, wearing long-sleeved clothing, and applying sunscreen. These variables were recorded as “always”, “most of the time”, “sometimes”, “rarely”, and “never” in the NHANES. The present analysis categorized the three variables as “rare” (never or rarely), “moderate” (sometimes), or “frequent” (always or most of time). Sun-protective behaviors were then classified as 0, 1, and 2–3 based on the number of behaviors with frequent use in three variables [15].

### Covariates

Covariates, including age (20–60 years), sex (men/women), race (non-Hispanic black, non-Hispanic white, Mexican American, other Hispanic and other race), marital status (single/never married, separated/divorced, widowed, married, and others), family poverty income ratio (PIR), body mass index (BMI), educational level (less than high school education/primary education, high school education/secondary education, and college education or above), smoking status, alcohol consumption, serum 25-hydroxyvitamin D [25(OH)D] level (severely deficient: < 25 nmol/mL, deficient: 25–49.9 nmol/mL, insufficient: 50–74.9 nmol/mL, and normal values: ≥ 75 nmol/mL) [16, 17], milk consumption, skin reaction, cardiovascular disease (CVD), diabetes mellitus, hypercholesterolemia, and weak/failing kidney. BMI is the body fat index for the weight (kg) to height (m^2^) ratio. PIR has been used in several studies as a marker of economic status [18]. Smoking status was categorized as current smoking (smoking every day or having more than 100 cigarettes over the course of their life), former smoking (currently non-smoking and having more than 100 cigarettes in the past), and never smoking (having fewer than 100 cigarettes) [19]. Drinking at least 12 alcoholic beverages of any type in the past year was defined as alcohol consumption [20]. Milk consumption was classified as a regular drinker (> 5 times per week), sometimes drinker (< 5 times per week), or never drinker (never drinking milk). Skin reaction to sun exposure without any protection after no sun exposure for several months was classified as nothing, mild, or severe. Participants with hypercholesterolemia were defined as individuals with high blood cholesterol levels. Participants with diabetes mellitus were diagnosed by a medical professional with hyperglycemia. Participants had a weak failing kidney if a physician diagnosed them with abnormal renal function. Participants with CVD were diagnosed by a physician with angina pectoris, heart attack, congestive heart failure, coronary heart disease, or stroke [21].

### Statistical analysis

R (http://www.R-project.org) was used to analyze the dataset. Categorical variables were expressed as percentages (%) and compared using χ2. Continuous variables were expressed as means ± standard deviation (SD) and compared using dependent-sample t-tests (normal distribution) or Kruskal–Wallis rank sum tests (non-normal distribution).

With sun-protective behaviors (0) as the reference category, multiple logistic regression models were utilized to evaluate the relationship between sun-protective behaviors and hypertension. Subgroup analyses were stratified by sex, race, and BMI. Three models were used in the present analysis: Model 1 (an unadjusted model), Model 2 (adjusted for age, sex, and race), and Model 3 (adjusted for model 2 and educational level, marital status, PIR, BMI, smoking status, alcohol consumption, milk consumption, serum 25-hydroxyvitamin D level, skin reaction, CVD, diabetes mellitus, hypercholesterolemia, and weak/failing kidney). When stratified by a certain variable, the model does not adjust this variable. We used multiple linear regression models to analyze the relationship of sun-protective behaviors and each sun-protective behavior with systolic and diastolic blood pressure, stratified by sex and race. Statistical significance was recognized by the value of *P* < 0.05.

## Results

### Description of the study population

The study analysis included data for 8,613 eligible participants (weighted *n* = 127,909,475), including 4,199 men and 4,414 women. The number of sun-protective behaviors with hypertensive participants was 1,964. The overall means (± SD) of systolic and diastolic blood pressure were 118.53 ± 14.36 and 71.60 ± 11.07. The mean age of study population was 39.01 ± 11.58 years. The percentages of sun-protective behaviors with 0, 1, and 2–3 were 47.7%, 36.8%, and 15.5%. The mean (± SD) of BMI was 28.97 ± 6.87 kg/m^2^. Race included Mexican American (*n* = 1,267, 9.3%), other Hispanic (*n* = 802, 6.1%), non-Hispanic white (*n* = 3,653, 65.1%), non-Hispanic black (*n* = 1,760, 11.5%), other race (*n* = 1,131, 8.0%). The demographic data characteristics organized by sun-protective behaviors status are represented in Table 1.

**Table 1:** Characteristics of participants stratified by sun-protective behaviors (NHANES 2009–2014)

**Variables**	**Sun-protective behaviors**				
	**0**	**1**	**2–3**	**Overall**	**P**
Number	4111(47.7)	3169(36.8)	1333(15.5)	8613(100)	
Hypertention, n (%)	882 (21.0)	775 (23.9)	307 (22.1)	1964 (22.2)	0.010
Systolic blood pressure, mmHg, Mean (SD)	118.91 (13.88)	118.47 (14.83)	117.54 (14.71)	118.53 (14.36)	0.014
Diastolic blood pressure, mmHg, Mean (SD)	71.64 (11.06)	71.68 (11.08)	71.31 (11.07)	71.60 (11.07)	0.576
Age, years, Mean (SD)	38.23 (11.76)	39.15 (11.40)	41.07 (11.10)	39.01 (11.58)	< 0.001
Sex, male, n (%)	2408 (59.8)	1353 (42.6)	438 (34.2)	4199 (49.6)	< 0.001
Race, n (%)					< 0.001
Mexican American	535 (8.3)	519 (10.5)	213 (9.8)	1267 (9.3)	
other Hispanic	337 (5.3)	307 (6.5)	158 (7.5)	802 (6.1)	
non-Hispanic white	1886 (67.5)	1218 (61.7)	549 (65.5)	3653 (65.1)	
non-Hispanic black	866 (11.8)	746 (13.4)	148 (6.2)	1760 (11.5)	
other race	487 (7.1)	379 (7.9)	265 (10.9)	1131 (8.0)	
PIR, Mean (SD)	2.37 (1.67)	2.44 (1.71)	2.69 (1.66)	2.44 (1.69)	< 0.001
25(OH)D, nmol/mL	58.80 (25.01)	56.94 (27.88)	57.95 (27.05)	57.98 (26.39)	< 0.001
Normal (≥ 75)	931 (28.4)	688 (30.8)	294 (30.7)	1913 (29.6)	
Insufficiency (50–74.9)	1630 (41.8)	1110 (35.9)	498 (36.9)	3238 (38.9)	
Deficiency (25–49.9)	1287 (25.5)	1106 (27.2)	448 (26.8)	2841 (26.3)	
Severe deficient (< 25)	263 (4.3)	265 (6.2)	93 (5.6)	621 (5.2)	
BMI, Kg/m^2^	28.90 (6.68)	29.29 (7.15)	28.45 (6.75)	28.97 (6.87)	0.014
BMI ≥ 30	1316 (31.0)	979 (32.5)	478 (35.7)	2773 (32.3)	
25 < BMI ≤ 30	1303 (33.3)	981 (31.5)	387 (31.8)	2671 (32.4)	
BMI ≤ 25	1492 (35.7)	1209 (36.0)	468 (32.5)	3169 (35.3)	
Marital status,married, n (%)	1890 (49.5)	1551 (54.2)	768 (62.0)	4209 (53.2)	< 0.001
Educational level, college or above, n (%)	2270 (60.7)	1811 (64.6)	913 (74.6)	4994 (64.3)	
Smoking status,current smoking, n (%)	653 (24.5)	395 (17.9)	108 (11.7)	1156 (20.1)	< 0.001
Alcohol consumption, n (%)	2996 (83.2)	2236 (81.7)	842 (78.2)	6074 (81.9)	< 0.001
Milk consumption,regular, n (%)	1713 (42.8)	1267 (42.5)	563 (43.0)	3543 (42.7)	0.195
Skin reaction,severe, n (%)	265 (8.0)	304 (13.0)	265 (25.2)	834 (12.5)	< 0.001
CVD, n (%)	161 (3.8)	141 (3.8)	38 (1.9)	340 (3.5)	0.042
Diabetes mellitus, n (%)	246 (4.9)	222 (5.7)	96 (6.2)	564 (5.4)	0.125
Hypercholesterolemia, n (%)	912 (27.7)	770 (29.6)	370 (31.2)	2052 (28.9)	0.023
Weak/Failing kidney, n (%)	62 (1.2)	49 (1.6)	26 (1.5)	137 (1.4)	0.517

Abbreviations: *BMI* Body mass index, *PIR* Poverty income ratio, *25(OH)D* 25-hydroxyvitamin D, *CVD* cardiovascular disease. Sun-protective behaviors were based on the number of behaviors with frequent use in three variables, including staying in the shade, wearing long sleeved clothing, and applying sunscreen. Values are presented as mean ± SD or number, (percentage of distribution)

### Association between Sun-protective behaviors and hypertension among different subgroups

Odds ratio (OR) was used as a measure of risk. Multiple logistic regression analysis was used to determine the association between sun-protective behaviors and hypertension (Table 2). Participants with sun-protective behaviors in the 2–3 category were prone to hypertension (OR = 1.29, 95% CI = 1.00–1.67, *P* = 0.0496) compared to those without sun-protective behaviors in Model 3. In the subgroup analysis of the fully-adjusted model (Table 2), we found that men, Mexican American, and BMI in the intermediate range who reported sun-protective behaviors (2–3) were prone to hypertension (OR = 1.57, 95% CI = 1.07–2.29, *P* = 0.0213; OR = 2.26, 95% CI = 1.09–4.74, *P* = 0.0294; OR = 1.78, 95% CI = 1.15–2.76, *P* = 0.0098).

**Table 2:** Association between Sun-protective behaviors and hypertension

		**Sun-protective behaviors**	
	**0**	**1**	**2–3**
**Total**			
Model 1	Ref	1.19(1.06,1.32)**	1.10(0.94,1.27)
Model 2	Ref	1.15(1.02,1.30)*	1.04(0.88,1.21)
Model 3	Ref	1.17(0.97,1.41)	1.29(1.00,1.67)*
**Stratified by sex**			
Women			
Model 1	Ref	1.33(1.13,1.56)***	1.08(0.89,1.32)
Model 2	Ref	1.31(1.10,1.56)**	1.04(0.83,1.29)
Model 3	Ref	1.40(1.05,1.87)*	1.14(0.80,1.64)
Men			
Model 1	Ref	1.08(0.92,1.26)	1.25(1.00,1.56)
Model 2	Ref	1.03(0.87,1.21)	1.12(0.87,1.43)
Model 3	Ref	1.00(0.77,1.30)	1.57(1.07,2.29)*
**Stratified by race**			
Mexican American			
Model 1	Ref	1.21(0.87,1.69)	1.68(1.12,2.50)*
Model 2	Ref	1.11(0.78,1.57)	1.46(0.94,2.25)
Model 3	Ref	0.96(0.53,1.76)	2.26(1.09,4.74)*
other Hispanic			
Model 1	Ref	1.32(0.89,1.95)	1.37(0.85,2.18)*
Model 2	Ref	1.19(0.79,1.80)	1.07(0.64,1.77)
Model 3	Ref	0.48(0.22,1.03)	1.00(0.38,2.59)
non-Hispanic white			
Model 1	Ref	1.03(0.86,1.22)**	1.03(0.82,1.29)
Model 2	Ref	1.01(0.84,1.22)	0.92(0.73,1.17)
Model 3	Ref	1.14(0.85,1.53)	1.28(0.87,1.86)
non-Hispanic black			
Model 1	Ref	1.59(1.28,1.96)***	2.06(1.44,2.94)
Model 2	Ref	1.56(1.24,1.97)***	1.67(1.13,2.45)**
Model 3	Ref	1.90(1.31,2.77)***	1.81(0.98,3.35)
other race			
Model 1	Ref	0.92(0.64,1.31)	0.67(0.43,1.01)
Model 2	Ref	0.99(0.68,1.43)	0.57(0.36,0.89)*
Model 3	Ref	1.20(0.62,2.36)	0.43(0.16,1.05)
**Stratified by BMI**			
BMI ≤ 25			
Model 1	Ref	1.01(0.77,1.32)	0.79(0.54,1.14)
Model 2	Ref	0.93(0.70,1.23)	0.68(0.45,1.00)
Model 3	Ref	0.99(0.61,1.60)	0.88(0.45,1.65)
25 < BMI ≤ 30			
Model 1	Ref	1.09(0.88,1.34)	1.25(0.95,1.64)
Model 2	Ref	1.16(0.93,1.45)	1.27(0.94,1.70)
Model 3	Ref	1.09(0.77,1.52)	1.78(1.15,2.76)**
BMI ≥ 30			
Model 1	Ref	1.28(1.10,1.50)**	1.24(1.00,1.53)
Model 2	Ref	1.28(1.08,1.52)**	1.17(0.92,1.48)
Model 3	Ref	1.26(0.96,1.64)	1.13(0.78,1.63)

Model 1: no covariates were adjusted

Model 2; age, sex, and race were adjusted

Model 3; age, sex, race, educational level, marital status, PIR, BMI, smoking status, alcohol consumption, milk consumption, serum 25-hydroxyvitamin D level, skin reaction, CVD, diabetes mellitus, hypercholesterolemia, and weak/failing kidney. When stratified by a certain variable, the model does not adjust this variable. BMI, Body mass index

^*^p < 0.05, **p < 0.01, ***p < 0.001

### Association between sun-protective behaviors with SBP and DBP

We also assessed the association of sun-protective behaviors with systolic and diastolic blood pressure using multiple linear regression analysis (Table 3). We found that sun-protective behaviors had no significant difference with systolic and diastolic blood pressure. The non-Hispanic white men who reported sun-protective behaviors (2–3) had positive association with systolic and diastolic blood pressure in the fully-adjusted model (95% CI = 1.30–7.40, *P* = 0.0052; 95% CI = 0.08–5.08, *P* = 0.0433).

**Table 3:** Association between sun-protective behaviors with SBP and DBP

	**SBP**			**DBP**		
	**Sun-protective behaviors**			**Sun-protective behaviors**		
	**0**	**1**	**2—3**	**0**	**1**	**2—3**
**Total**	Ref	0.33(-0.67,1.34)	0.62(-0.75,1.99)	Ref	0.32(-0.45,1.10)	-0.14(-1.20,0.92)
**Stratified by sex and race**						
Men						
Mexican American	Ref	-0.74(-5.12,3.64)	3.69(-3.41,10.79)	Ref	-0.27(-3.71,3.16)	-2.44(-8.01,3.14)
other Hispanic	Ref	-1.56(-6.33,3.21)	0.74(-6.89,8.38)	Ref	-2.27(-5.83,1.29)	1.44(-4.25,7.14)
non-Hispanic white	Ref	1.33(-0.75,3.41)	4.35(1.30,7.40)**	Ref	0.46(-1.25,2.17)	2.58(0.08,5.08)*
non-Hispanic black	Ref	2.02(-1.32,5.35)	-3.19(-9.29,2.91)	Ref	0.76(-1.93, 3.45)	-1.03(-5.95,3.88)
other race	Ref	-1.44(-5.32,2.44)	-4.25(-9.35,0.86)	Ref	0.35(-2.73,3.43)	-1.24(-5.29,2.81)
Women						
Mexican American	Ref	0.61(-3.77,4.99)	-0.20(-5.39,4.99)	Ref	1.56(-1.83,4.96)	-0.71(-4.73,3.32)
other Hispanic	Ref	0.81(-4.35,5.97)	0.68(-5.40,6.77)	Ref	-2.04(-5.67,1.59)	-3.56(-7.85,0.72)
non-Hispanic white	Ref	-0.66(-2.72,1.41)	-0.12(-2.64,2.39)	Ref	0.04(-1.51,1.59)	0.21(-1.68,2.09)
non-Hispanic black	Ref	1.35(-1.91,4.60)	1.84(-3.51,7.19)	Ref	1.62(-0.85,4.10)	-1.22(-5.29,2.85)
other race	Ref	1.41(-3.02,5.85)	-1.50(-6.25,3.24)	Ref	1.10(-2.41,4.62)	-0.92(-4.68,2.84)

Model 3; age, sex, race, educational level, marital status, PIR, BMI, smoking status, alcohol consumption, milk consumption, serum 25-hydroxyvitamin D level, skin reaction, CVD, diabetes mellitus, hypercholesterolemia, and weak/failing kidney

When stratified by a certain variable, the model does not adjust this variable

*SBP *Systolic blood pressure, *DBP *Diastolic blood pressure

^*^p < 0.05, **p < 0.01, ***p < 0.001

### Association between each sun-protective behavior with SBP and DBP

Additional multiple linear regression analysis was used to assess the association of each sun-protective behavior with systolic and diastolic blood pressure (see Additional file 1). In the fully-adjusted model, we found that the association between other-Hispanic men with frequent wearing long-sleeved clothing and diastolic blood pressure was positively correlated (95% CI = -0.69–10.34, *P* = 0.0253).

## Discussion

In previous studies, opländer study confirmed that ultraviolet A (UVA) radiation was associated with lower blood pressure [22]. Feelisch and coworkers indicated that the drop in blood pressure was due to arterial vasodilatation caused by ultraviolet A (UVA) radiation and found that the skin can mobilize nitric oxide through photolysis of cutaneous nitrite or nitrate to lower blood pressure with UVA radiation [23]. Ultraviolet A (UVA) radiation is derived from sunlight and sun-protective behaviors cause a reduction in UVA absorption [24], which made us explore the relationship between sun-protective behaviors and hypertension. Finally, we conducted this study and found a positive association between sun-protective behaviors and hypertension.

Sun-protective behaviors are different for sexes. Pinault et al. found that sun-protective behaviors in females are more significant in the summer months for Canadian adults [25]. And different races have different awareness of sun protection because of factors such as culture, geographical location, and so on [26]. For people with different BMI, the larger the body surface area will receive more sunlight [27]. Therefore, we performed subgroup analyses using these three variables. The results showed that sun-protective behaviors were associated with an increased risk of hypertension, which was more significant in sex and race. The analysis of people with different BMI suggests that those in the intermediate range are at greater risk of developing hypertension. In high BMI, more sunlight absorption can lower blood pressure, but high BMI increases the risk of insulin resistance [28], which could increase the risk of developing hypertension [29]. There is currently no relevant literature discussing this.

To acknowledge the relationship of sun-protective behaviors with systolic and diastolic blood pressure stratified by sex and race, we performed a multiple linear regression and found no significant differences. Because systolic and diastolic blood pressure data were collected after antihypertensive medication or life interventions, which may have contributed to the fact that sun-protective behaviors were not associated with systolic and diastolic blood pressure. And it was difficult to obtain pre-intervention systolic and diastolic blood pressure due to the limited database. However, even with post-intervention data, we found significant differences between non-Hispanic white men who reported sun-protective behaviors (2–3) with diastolic and systolic blood pressure, suggesting that sun protection programs should be more important for this population.

In our study, analysis of each sun-protective behavior and hypertension combination is shown in Additional file 1. After adjusting for confounders, the outcomes showed that the association between other race men who reported wearing long-sleeved clothing frequently and diastolic blood pressure was positively correlated. Staying in the shade and applying sunscreen were not associated with increased risk of hypertension. There are no relevant studies examining the relationship between each sun-protective behavior and hypertension, and more study is needed to verify the results.

Hypertension causes a great number of CVD morbidity and mortality cases [30]. Decreasing blood pressure levels has a significant influence on reducing the societal disease burden [31]. The present study adjusted for several potential confounders in the NHANES to analyze the association between sun-protective behaviors and hypertension, confirming that individuals with sun-protective behaviors are more likely to have hypertension. In the future, patients will be able to improve their blood pressure by following appropriate sun-protective behavior recommendations provided by their medical practitioners.

There were several limitations in the present study. Firstly, the cross-sectional nature of the study introduced intrinsic limitations. The data from the NHANES were obtained between 2009 and 2014. In the future, a randomized controlled study will be necessary to verify these results. Secondly, the history of hypertension and sun-protective behaviors were self-reported, resulting in under- or over-reported questionnaire outcomes. Thirdly, due to the limited database, the systolic and diastolic blood pressure data were obtained post-intervention. This may lead to differences in results. Finally, the participants were 20–60-years-old. Given that hypertension often occurs in the elderly, future research will need to focus on patients older than 60 years.

## Conclusion

The present cross-sectional study results demonstrated that sun-protective behaviors of the 2–3 category were associated with increased risk of hypertension, but not with higher systolic and diastolic blood pressure. We only found that non-Hispanic white men who reported sun-protective behaviors (2–3) were positively associated with systolic and diastolic pressure. Consequently, patients should be encouraged to avoid excessive sun protection due to the high risk of hypertension and sun-protective behaviors should be sensible for non-Hispanic white men. Future clinical studies could focus on developing specific sun protection programs for different populations.

### Supplementary Information

**Additional file 1.** Association between each sun-protective behavior with SBP and DBP.

## Data Availability

The dataset supporting the conclusions of this article is available in the NHANES repository, https://www.cdc.gov/nchs/nhanes.

## Abbreviations

NHANES: National Health and Nutritional Examination Survey
CVDs: Cardiovascular diseases
CVD: Cardiovascular disease
PIR: Family poverty income ratio
BMI: Body mass index
25(OH)D: Serum 25-hydroxyvitamin D
UVA: Ultraviolet A
SBP: systolic blood pressure
DBP: diastolic blood pressure
